# Leading across cultures in the human age: an empirical investigation of intercultural competency among global leaders

**DOI:** 10.1186/2193-1801-3-127

**Published:** 2014-03-06

**Authors:** Michael F Tucker, Ron Bonial, Adam Vanhove, Uma Kedharnath

**Affiliations:** Tucker International, 5777 Central Ave., Suite 110, Boulder, CO 80301 USA; Colorado State University, 5777 Central Ave., Suite 110, Boulder, CO 80301 USA

## Abstract

This article reports on a major, large-scale two-year empirical study to investigate intercultural competencies among global leaders and the relationship of these competencies to criteria of high performance global leadership. The study was designed to contribute to the emerging field of global leadership research by identifying and measuring proximal attributes and leadership criteria as suggested by Zaccaro’s trait-based leadership model (American Psychologist 62: 6-16, 2007). Only global leaders were included in the study – CEO’s, General Managers, Function Heads, or those with Profit and Loss responsibility for their businesses. These 1867 leaders of 13 nationalities were engaged in leading people across cultures – either on international assignment or working from their home base. A set of six intercultural competencies and three criteria of global leadership success were identified and compared across nationalities. The competencies were measured and used to predict success over time. Applications are discussed in terms of global leadership assessment and development.

## Background

The emergence of globalization just a short time ago has become the norm. Companies adept at identifying business opportunities anywhere in the world and effectively deploying resources to capture those opportunities are enjoying unprecedented success. The days are gone when a major company can be complacent by being successful in its home market, or even in one or two cross-border markets. Even if a major company chooses not to expand globally, international competitors will enter its favorite markets. Among the challenges facing these companies are competing in a global environment and leadership talent.

To be successful, global organizations need leaders who can drive business on a global scale. As we enter the Human Age, where Talentism is the new Capitalism, no organization can afford to overlook optimizing the performance of leaders who operate globally. Productivity and innovation in the Human Age require talent strategies that focus on developing and nurturing global leaders.

Leading across cultures is a critical element of leading in the Human Age and unleashing the power of what is humanly possible. It often requires making decisions in complex or ambitious environments, understanding cultural nuances and adapting one’s style accordingly. A good track record in one country does not guarantee success in the global arena, nor will merely exposing high-performing leaders to new cultures make them effective multinational leaders.

Recent studies by IBM of 1500 CEOs and DDI of 14,320 HR professionals and business leaders show that a majority of companies do not have the leaders needed to keep up with the speed of business, are not satisfied with the quality of their leaders (particularly Asian leaders), and do not have the bench strength to meet future business needs (IBM 2011; Boatman & Wellins 2011).

These two surveys are concerned with leadership in general. The situation with global leadership talent is even more dire. Researchers have argued that global leadership is more complex than domestic leadership in that leadership responsibilities and issues span across cultures (e.g., ethical challenges, team building, dealing with different perspectives) (Osland & Bird 2006). Very few companies, indeed, would say that they are satisfied with leaders’ current proficiency in this type of leadership in their organizations.

There is a growing awareness that a new kind of leadership is emerging to show the way. Perhaps this was best stated by the Conference Board: “The pace of globalizing business strategy is staggering. Successful implementation of strategy, however, by interculturally competent people, supported by appropriate corporate practice, is the greatest need and the key to success”. An article written by Joann S. Lublin in the Wall Street Journal stated: “Global businesses are looking for leaders who have the ability to move easily between different cultures… Finding such executives is very challenging… The talent pool is very small” (Lublin 2011, page B1). A study by Right Management and the Chally Group found that some 80% of HR professionals rated Cultural Assimilation as the greatest challenge facing successful leaders outside of their home country (Right Management 2011). A major study found that “Cultural issues will dominate the new competencies that will be required for global leaders over the next ten years” (Training Magazine ).2011). Jim Collins, in his book *Good to Great* (Collins 2001) provided powerful support to the idea of people first. He found that the first step in taking a good company to a great one was not strategy, but “getting the right people on the bus, in the right seats, and then figuring out how to take the business someplace great”. In the global business environment, the international bus must be led by people who can perform at high levels across cultures.

Although the field of global leadership research is new and emerging (Mendenhall et al. 2008) a number of studies have been reported in recent years. Summaries of these appear in Dickson, Den Hartog, and Mitchelson (2003), House, Wright and Aditya (1997), Dorfman (2003), Peterson and Hunt (1997), and Mendenhall et al. (2008). These studies, taken together, have included a large number of leadership competencies (perhaps over 50).

The early literature on global leadership historically focused on U.S. samples without carefully testing whether the research findings generalize across cultures outside the U.S. Over the past twenty years, researchers have started to test and validate theories and models of global leadership across cultures. This allows them to identify the capabilities needed for successful global leadership with more accuracy.

For example, Kuhlmann and Stahl (1998) studied expatriates to determine the competencies that predict their effectiveness (reported in Stahl 2001). They found that seven competencies are needed for global leaders to be successful including:

- Tolerance for ambiguity,
- Behavioral flexibility,
- Goal orientation,
- Sociability,
- Empathy,
- Nonjudgmentalness, and
- Meta-communication skills.

House, Javidan, Hanges and Dorfman (2002) reported the results of their Global Leadership and Organizational Behavior Effectiveness (GLOBE) project, in which they studied leaders spanning 61 nations to find globally universal leadership competencies. They found support for nine global leadership competencies:

- Uncertainty avoidance (degree to which people rely on norms, rituals)
- Power distance (degree to which power is equally shared)
- Societal emphasis on collectivism (degree to which norms and practices reward collective distribution of resources)
- Family organizational collectivistic practices (degree to which individuals express pride, loyalty, and cohesiveness in families or orgs)
- Gender egalitarianism (degree to which gender role differences are minimized)
- Assertiveness (degree to which individuals are assertive in social relationships)
- Future orientation (degree to which individuals invest in the future)
- Performance orientation (degree to which people are rewarded for performance improvement/excellence)
- Humane orientation (degree to which people are rewarded for being friendly, caring, kind to others)

More recently, McCall and Hollenbeck (2002) found support for seven global competencies after they surveyed global leaders across 36 countries. They found support for the following competencies:

- Flexibility in strategy and tactics,
- Cultural sensitivity,
- Ability to deal with complexity,
- Resilience and resourcefulness,
- Honesty and integrity,
- Personal stability, and
- Sound technical skills.

This literature supports the need for several areas of investigation that are addressed in the present study:

- The need to study a representative sample comprised exclusively of global leaders.
- The need for a concise set of intercultural competencies and a separate set of global leadership success factors with good psychometric properties that can be used to compare among leaders of different nationalities.
- The need to validate intercultural competencies against separate criteria of global leadership success.
- The need to detect social desirability or “fake good” responses in self-response instruments and make appropriate corrections.
- In order to meet these needs, the following questions were addressed in the study:
- What are the intercultural competencies among global leaders?
- What are the strongest of these competencies and those in need of greatest development?
- How do these competencies differ across nationalities?
- What are the areas of greatest leadership success?
- How do these success factors differ across nationalities?
- Which competencies are most strongly predictive of leadership success?

### Intercultural competence theory and model

#### Competency standards

We agree with Bird (2008, p. 65) that “there are three clear standards that must be met to define an individual characteristic or capacity as a competency: (1) it must exist prior to performance; (2) it must be causally linked to performance; and (3) it must be possessed by superior, but not by average or subpar, performers.” These standards guided our research method. We assessed competencies prior to assessing performance; we linked competencies to performance, and; we predicted membership in the top 20% of our leadership sample on performance factors.

#### Competency definition and theoretical model

We agree with the definition of global leadership competencies as stated by Jokinen (2005) “those universal qualities that enable individuals to perform their job outside their own nationality as well as organizational culture, no matter what their educational or ethnic background is, what functional area their job description represents, or what organization they come from.”

**Figure 1:**
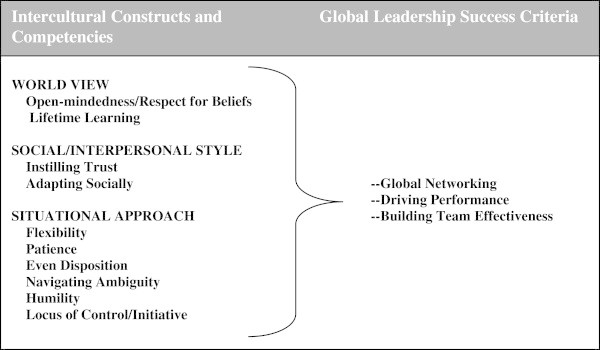
**Transcultural leadership model.**

As stated earlier, over 50 leadership competencies (Jokinen’s “Universal Qualities”) have been studied. As a starting point for our study, we began with the Tucker, et al. (2004) study in which 14 intercultural competencies were measured among 2131 corporate expatriates representing 11 nationalities (many of whom were leaders) and used these competencies to predict separate criteria of intercultural adjustment among 157 of these expatriates. Intercultural adjustment was in turn correlated with an expatriate job performance scale. The 2004 Tucker et al. study was based on a theoretical model that emerged over 30 years of empirical field research (Yellen and Mumford (1975); Tucker et al. (1978); Hawes and Kealey (1981); Tucker (1982); Tucker (1983); Tucker, (1994)); The model provided a framework for defining and measuring intercultural adjustment among expatriates and predicting it from antecedent intercultural competencies. We examined this model for logical application to global leaders, modified and added to it based on the literature, and settled on a new model that we call Transcultural Leadership, which is illustrated in Figure 1 below and then described.

### Intercultural constructs and competencies

#### World view

Leadership behavior that demonstrates an openness to diverse ways of people and their beliefs, and a commitment to global learning.

##### Open-mindedness/respecting beliefs

Open-minded individuals are receptive to different beliefs and ideas without feeling challenged or threatened. Those with the attitude that their own or their nation’s way is inherently superior face difficulties in working internationally. According to Seelye (1996), “The beginning of wisdom is the ability to see at least two sides of a story,” Rhinesmith (1993) said “The first lesson in an international assignment is that your perspective is just one side of the elephant. To adjust to the new culture and be effective, you have to be willing to crawl around the elephant, understand how it looks from all sides, and be able to communicate and empathize with the people who are looking at it from the other side.” This competency includes the capacity to be non-judgmental of others’ spiritual and political beliefs. According to Harris and Moran (1991), “The ability to express respect for another is an important part of effective relations in every country. All people like to believe and feel that others respect them, their ideas and their accomplishments” Harris and Moran (1991). Global leaders who demonstrate a willingness and ability to respect and be interested in beliefs of other cultures are more likely to establish meaningful intercultural relationships.

##### Lifetime learning

A deep knowledge of other nations and cultures is one of the factors discovered by Tucker et al. (2004) that “differentiates those who successfully adapt to working with other cultures.” This involves interest in cultural history and tradition as well as current local events. Walsch, Heyman, & Devaney (2008) “The ability to gain this knowledge is characteristic of those who are committed to a pattern of lifetime learning. This pattern is also important for one’s career.”

#### Social/interpersonal style

Connecting well with different kinds of people in business and social environments.

##### Instilling trust

There are many benefits for the person who maintains an attitude of trust in other people. “The high-trusting person is less likely to be unhappy, conflicted, or maladjusted; he or she is liked more and is sought out as a friend more often, both by low-trusting and high-trusting others” Rotter (1980). According to Covey (2006), page 286 “The first job of a leader is to inspire trust. The ability to do so, in fact, is a prime differentiator between a manager and a leader. To inspire trust is to create the foundation upon which all truly successful enterprises – and relationships – stand.” Furthermore, quoting from Black, Gregerson & Mendenhall (1992), “Trusting employees and involving them in the decision-making process results in better overall decisions, greater acceptance of decisions, and increased satisfaction in international leadership situations.” Also, according to Tucker (2008); Cleland and Ireland (2002), “The ability to develop trust among team members is an important aspect of international team performance.”

##### Adapting socially

This reflects the ability to socialize with new people in unfamiliar social situations and to be accepted by new groups of people. Teagarden and Gordon (1995) explain:

“Possession of technical skills may not be sufficient for successful adaptation or for information transfer, which is often considered a key strategic objective. The literature suggests that relationship skills are also important. One study found that caring about coworkers and being considerate of others predicted ability to transfer knowledge to host nationals…Still others have found that knowledge of people of other cultures, willingness to interact with them, and positive attitudes toward them are indispensible to adjustment and intercultural interaction.”

Business goals are accomplished all over the world in social situations, often informally. One who feels comfortable only in small, intimate groups may feel lost in new and unfamiliar settings. This competency also includes the demonstration of interest in other people. The importance of social adaptability and interpersonal interest was stated by Kohls (1996): “Much of your effectiveness on the job and satisfaction will depend on how well you build working and social relationships with the host nationals.” Those who are sincerely interested in, accepting of, and concerned for others, have a great advantage in adjusting to people of other cultures.

#### Situational approach

Leadership style that works well in situations where different cultural values and attitudes come into play.

##### Flexibility

Flexibility when working with people from other cultures is important because we all view problems and situations through thick cultural lenses. There is always more than one valid way of approaching and solving a problem; the approach and solution that we prefer is largely a matter of our cultural lens. Kohls (1996) states: “We are doomed to carry our complete load of cultural baggage wherever we go. There will be no stripping down to lighten the burden or to make the trip easier. It’s important, therefore, to know as much as possible about what our culture has packed for us to carry endlessly about the world.” Guy and Mattock (1996) explain “When your preparation is complete, the great thing is to be ready to adapt your methods to the local terrain. Flexible responses are part and parcel of good tactics.”

The ability to consider new ideas and to acknowledge there are multiple ways to approach and solve problems is necessary for effective global leadership. Flexibility also requires exploring new ways of doing things. The willingness to take risks, meet challenges and cope with change greatly enhances global leadership.

##### Patience

“Time” differs by culture. Failure to understand this may lead to frustration from unexpected delays or seemingly rash decisions. Leaders must remain patient when local business protocol demands a decision-making process or way of doing business that is unique to a certain culture. In his classic book *The Silent Language* Hall (1959), explained how “time is perceived and managed differently across cultures and how patience is required to deal with these differences.” Nisbitt (2003) provided a deep and thorough explanation of and description of how Asians and Westerners think differently and why. The two fundamentally different ways of thinking require a large amount of patience in order for Asians and Westerners to relate well and work together. Aislein and Mastrin (2001) describe the French Cartesian style of thinking and decision-making, which suggests patient responses of the French to work with other cultures and vice-versa.

##### Even disposition

Leading authors on the nature of leadership, Kouzes & Posner (1995); Dotlich, Cairo & Rhinesmith (2009); Zenger Folkman (2009), include effective interpersonal skills and understanding one’s style and effect on others as core competencies.. In an international environment, this means remaining calm, not being critical of oneself and exhibiting a good sense of humor. The ability to bring humor into difficult or confusing situations and to learn from one’s own mistakes often helps to ease tensions and facilitate communication across cultures. As Kohls (1996) explains: “A sense of humor is important because there is going to be much to weep or get angry or annoyed or embarrassed or discouraged about. No matter how many of the other important cross-cultural skills you have, the ability to laugh things off will be the ultimate weapon against despair.” Doskoch (1996) summarized associations between humor and laughter on one hand, and an amazing variety of mental and physical health benefits on the other. These included a positive mood in the face of stress, relaxation, belonging and social cohesion, creativity, and even enhanced physical immunity*.*

##### Navigating ambiguity

Global leaders who tolerate and successfully deal with ambiguity are able to see through vagueness and uncertainty, eventually figuring out the ways of a foreign culture. They are not threatened by ambiguity or seek “black or white” solutions, but enjoy dealing with the unknown. Rhinesmith (1993) listed “the ability to feel comfortable with ambiguity” as one of the basic capacities of a global mindset. Rhinesmith continued to say,

“Global management is more complex because one faces the challenge of managing increased ambiguity in decision-making. This results from being exposed to many more variables and broader issues, which often have philosophical, moral and cultural dimensions, as well as business considerations. This makes the decision process more ambiguous.”

##### Humility

Successful global leaders engage in the processes of adjustment and overcoming challenges with a sense of humility. They realize that an egotistical, self-centered, arrogant approach is quickly rejected. People around the world appreciate leaders who ask for help, advice and information, instead of assuming that they already know as much as they need to know. Collins (2001) discussed a surprisingly consistent characteristic of leaders who took their organizations from being good to being great. Called “Level 5 Leadership”, this is defined as building enduring greatness through a paradoxical blend of personal humility and professional will.

##### Locus of control

“Locus of control refers to the extent to which individuals believe that they can take the initiative and control events that affect them” Rotter (1966); Ward & Kennedy (1992), Ward and Kennedy (1993). Individuals who have an internal locus of control believe that the events in their lives are generally the result of their own behavior and actions and they take responsibility for their actions. On the other hand, individuals who have an external locus of control believe that the events in their lives are generally determined by chance, fate, circumstance or other people.

### Leadership theory

**Figure 2:**
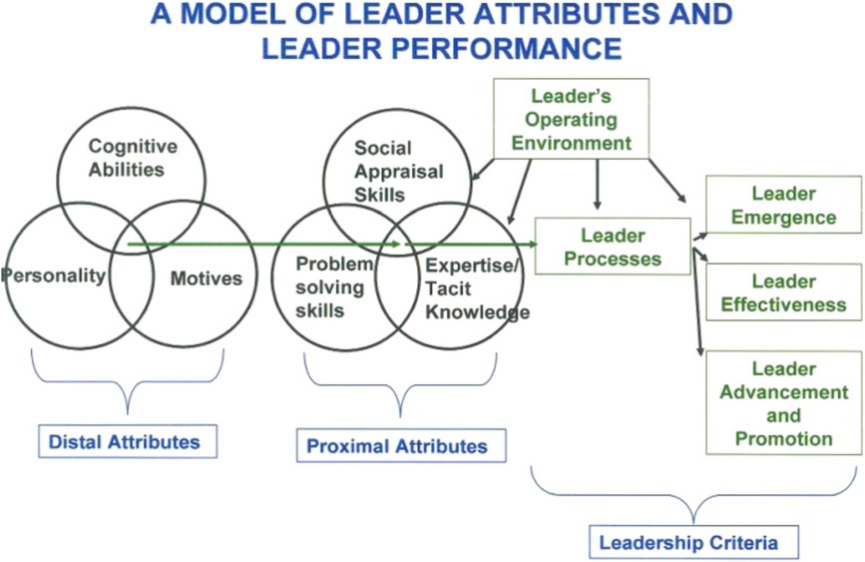
**Leader performance model.** Taken from: Zaccaro 2007.

Leadership theory has focused on the traits of a leader, the attributes that a leader applies, and the situation in which leadership behavior occurs (see special issue in American Psychologist, Volume 62, Issue 1, 2007). Zaccaro (2007) offered the following trait-based model of effective leadership (Figure 2) wherein distal leader traits (distal attributes) affect proximal attributes, and together these attributes influence leader processes (moderated by operating environment), which in turn influences indicators of leader performance. Given that the field of global leadership remains in its infancy (Bird 2008), the study reported here adopts this general trait-based model of leader effectiveness, applies it to global leadership effectiveness, and attempts to identify a more precise set of proximal attributes specifically relevant to global leadership. In addition, the present study attempts to establish an efficient set of global leader effectiveness criteria.

#### Global leader success criteria

In order to meet the standard of linking intercultural competencies to performance, we settled on three performance areas, or global leader success criteria as follows:

##### Global networking

This criterion is defined as:

- Developing a network of international relationships.
- Making successful transitions to working with people of other nationalities.

##### Driving performance

This area includes:

- Evidence of effectiveness in a global leadership role.
- Team achievement of global business goals and company success in countries of operation.
- The company being seen as a preferred place to work by the local workforce.

##### Building team effectiveness

This area includes:

- Successful coaching of team members and developing competency.
- Building trust and a culture of respect.
- Learning from the team.

#### Theoretical statement

##### Our theoretical statement is as follows

Intercultural competencies have a significant influence on global leadership success. There is a set of ten attributes (intercultural competencies) among global leaders that can be measured on a universal (etic) level and used on a culturally contingent (emic) level to compare competencies across cultures and to predict three separate criteria of global leadership success.

#### Hypotheses

The hypotheses to be tested were the following:

H1Responses from global leaders to an intercultural competency assessment instrument will yield a set of factors with acceptable psychometric properties to confirm the ten proposed competencies.

H2The extracted factors will assess the strengths and weaknesses of intercultural competencies among global leaders.

H3The extracted factors will compare the strengths and weaknesses of intercultural competencies across nationalities of global leaders.

H4Reponses from global leaders to an instrument assessing criteria of leadership success will yield a set of factors with acceptable psychometric properties to confirm three proposed success areas.

H5The extracted criterion factors will assess the level of success among global leaders.

H6The extracted criterion factors will compare success across nationalities of global leaders.

H7The extracted intercultural competency factors will predict the success criterion factors at acceptable levels of confidence.

## Method

### Intercultural competency assessment with the global leader TAP

The GLTAP is a 107-item instrument, designed to assess the ten intercultural competencies in our model, including nine social desirability, or “fake good” items. A five-point, Likert-type scale was used to measure responses to each item (Likert 1932). The response anchors ranged from “strongly agree” to “strong disagree”.

### Assessment of global leadership success with the Survey of Global Business Experience (GBE)

This phase of the study was designed to address the criterion problem in assessment prediction research. As stated earlier, there have been a large number of leadership competencies identified and studied, but few studies have shown that these competencies lead to and predict leadership success criteria. Quoting from Guion (1998), p. 130 “The criterion problem continues to lead all other topics in lip service and to trail most in terms of work reported,” and “Improved prediction cannot be expected without firm understanding of what we want to predict.”

In order to address this criterion problem, leaders who had earlier completed the GLTAP completed a 12-item instrument called the “Survey of Global Business Experience” (GBE). A 20-point scale was used to measure these items, so that an item could be rated from 0 (complete lack of agreement) to 20 (compete agreement). “This scale therefore has a meaningful absolute zero point” Guilford (1965).

### Participants

The GLTAP and GBE were completed on-line between April of 2010 and September of 2011. A total of 1953 leaders from a wide range of nations completed the GLTAP. Of those leaders, 1867 represented the 13 nations examined in this study. A total of 834 individuals from these 13 nations subsequently completed the GBE. Table 1 shows sample sizes for each of the 13 nations examined in this study.

**Table 1:** **Total sample size by nationality for GLTAP and GBE data**

Nationality	GLTAP ( *N* )	GLTAP and GBE ( *N* )
Australian	189	76
Belgian	93	40
Brazilian	140	54
Canadian	192	103
Chinese	122	50
French	178	94
German	173	104
Indian	186	85
Japanese	139	41
Norwegian	67	18
Swedish	52	32
United Kingdom	175	79
American	161	58
Total *N*	1867	834

Jokinen’s (2005) definition of global leadership competencies stated earlier guided our selection of study participants. We screened for participants who were leading across cultures, and working in many different industries. We were not concerned about different levels of responsibility.

A wide variety of organizations were included, from mid-size to Fortune 100 organizations. There were 134 industries represented. Some 66% of respondents were male, 34% female (mean age = 43). Only global leaders were included in the study. Some 80 NASA International Project Managers and their international colleagues were included. We defined a global leader as one who is engaged in managing people across cultures either on international assignment or working from a home base. Responsibilities for leaders included (respondents were asked to check all that applied):

- 59% as having top executive responsibility;
- 51% as having profit and loss responsibility;
- 50% as having responsibility over a group of businesses;
- 42% as having top executive responsibility for their business function.

### Analyses of GLTAP responses

First, means were calculated on the GLTAP *Social Desirability* scale, and MANOVAs were conducted to determine if *Social Desirability* affected scores on the GLTAP competency scales (high *Social Desirability* scores may lead to systematically erroneous high competency scores). Next, confirmatory factor analyses (CFA) were performed on (a) the English-as-a-first language samples, and (b) the total sample in order to test the hypothesized ten factor structure. Lastly, another set of CFAs were conducted to examine the factor structure.

### GLTAP intercultural competency strengths and weaknesses and comparison across nationalities

Once the GLTAP factor structure was established, measurement equivalence analyses were performed to establish (a) how well the GLTAP model fit each national sample, separately, and (b) how well the GLTAP items functioned across nationalities in order for us to gain confidence the nationalities could be compared. These analyses were done by means of CFA. First we examined model fit, separately, for each of the 13 national samples. Then, we conducted a multiple groups CFA to examine configural invariance across national samples.

### Analyses of GBE responses

Confirmatory factor analyses were performed on the GBE responses to study the hypothesized three criterion success factors.

### GBE success strengths and weaknesses and comparison across nationalities

Measurement equivalent analyses were done to determine if the GBE success factors could be compared across the nine nationalities. A factor model, along with a combined score, was used to estimate global leadership success for the total sample as well as to compare success across these nationalities. Major differences were found in the rank orders of the nationalities for the intercultural competencies as compared with the rank orders of success factors. In order to explain the results of the success factors across nationalities, a rank-order correlation was calculated for the success factor ranks and the Hofstede Power Distance Rankings (Hofstede 1997).

### Predicting leadership success

This final step of the study method was to attempt the prediction of GBE global leadership success with the GLTAP intercultural competencies, which had been measured at an earlier point in time. Total scores on the competency factors were correlated with total scores on the success factors. An omnibus ANOVA, a test of whether all competency factor scores together predicted success scores, was performed.

A final, extreme groups analysis was conducted, whereby those who scored in the top 20% and the bottom 20% on the GBE success factors were identified. An Omnibus Test of Model Coefficients was used to predict membership in these two groups by means of the GLTAP competency factors.

## Results

### Social desirability

A series of ANOVAs were conducted to examine whether individuals differed in their responses to items across the ten hypothesized factors based on social desirability scores. Cutoffs of the top 10%, 15%, and 20% were used to compare to the responses of those in the remainder of the sample. No significant differences were found using any of the cutoffs. Thus, no participant data was dropped for further analyses based on social desirability scale scores.

### Analyses of GLTAP responses

#### Confirmatory factor analysis (CFA)

Using all 98 items comprising ten hypothesized factors (social desirability items were excluded), a confirmatory factor analysis (CFA) was conducted across the English-as-a-first-language samples (i.e., Australia, Canada, United Kingdom, and United States; *n* = 717). This CFA did not converge, resulting in no fit statistics for the analysis. This same analysis was then conducted across the entire participant sample (*N* = 1,953), but again the ten factor CFA did not converge. Based on the item factor loadings and a content reanalysis we retained 51 of the original 98 items to create a more parsimonious six factor structure. We identified these factors as *Respecting Beliefs* (seven items; α = .82), *Navigating Ambiguity* (nine items; α = .80), *Instilling Trust* (eight items; α = .72), *Adapting Socially* (12 items; α = .86), *Even Disposition* (seven items; α = .72), and *Demonstrating Creativity* (eight items; α *=* .72*)*.

We then used CFA to assess the six factor GLTAP structure. Across the entire sample (*N* = 1,953), the six factor structure did not fit the data well, *χ*^*2*^ (1024) = 9398.87, *CFI* = .70, *TLI* = .68, *RMSEA* = .07, according to CFI and TLI (>.90) and RMSEA (<.08) fit standards suggested by MacDonald and Ho (2002). Based on these criteria, the six factor model did not fit the data well. In exploring the factor loadings of the six factor model it became clear items in the *Adapting Socially, Even Disposition, and Demonstrating Creativity* factors were not functioning as expected. In addition, one item from the *Instilling Trust* factor fit poorly with the other items under that factor, and was dropped from subsequent analyses. This left 23 items across three factors: *Respecting Beliefs, Navigating Ambiguity,* and *Instilling Trust*. The three-factor model, which included a higher-order “global leadership” factor, fit the data substantially better, *χ*^*2*^ (227) = 1608.48, *CFI* = .89, *TLI* = .88, *RMSEA* = .06, and approached acceptable fit according to MacDonald and Ho’s (2002) standards. In addition, each of the three factors loaded highly onto the higher-order factor—Respecting Beliefs = 0.79, Navigating Ambiguity = 0.84, and Instilling Trust = 0.87—suggesting that each was a relatively strong indicator of the higher-order construct of global leadership. We were unable to find support for Hypothesis 1 in that the proposed 10 factor model did not demonstrate acceptable model fit. However, we were able to identify a more parsimonious alternative model that demonstrated improved fit.

#### Measurement equivalence for the three factor GLTAP model

Next, we tested whether the three factor GLTAP model could be meaningfully compared across nationalities. We did so by conducting measurement equivalence analyses. First, we conducted a CFA of the three factor model for each national sample separately. This gave us a better understanding of how well the three factor model held up across each nationality. Second, we conducted a multiple group CFA to test for configural invariance. Here, factor loadings associated with each national sample were compared, with model fit indices indicating congruency across each nationality’s factor loadings.

The results of the separate CFAs by nationality are shown in Table 2. Clearly, the data fit better among some nationalities than others. Nationalities that showed relatively better fit include: Australia, Canada, China, France, Germany, India, and the United Kingdom. Nationalities that showed relatively worse fit were Brazil, Japan, Norway, and Sweden. In the cases of the Norwegian and Swedish samples, a lack of power due to small sample sizes may have been the cause of poor fit. Surprisingly, model fit among the American sample was also lower than expected (only the RMSEA value met acceptable fit standards).

**Table 2:** **CFAs for each national sample on the GLTAP 3-factor model**

Nationality	N	CFI	TLI	RMSEA
Australian	189	.86	.84	.07
Belgian	93	.70	.67	.07
Brazilian	140	.41	.35	.14
Canadian	192	.83	.81	.07
Chinese	122	.81	.79	.07
French	178	.85	.84	.06
German	173	.84	.83	.07
Indian	186	.79	.77	.07
Japanese	139	.60	.55	.10
Norwegian	67	.60	.56	.11
Swedish	52	.65	.62	.13
British	175	.84	.82	.06
American	161	.69	.65	.07

After examining the results of the separate CFAs by nationality, we then conducted two multiple-group CFA models to test for configural invariance. The first model included all 13 nationalities (Model 1). Likely due to a lack of statistical power and the poor model fit found for a number of the nationalities at the previous step, Model 1 did not converge. In an attempt to remedy the latter, the nationalities with the poorest model fit, as indicated by the fit statistics shown in Table 2 (Brazilian, Japanese, Norwegian, and Swedish samples), were not included in Model 2. Model 2, which included the remaining nine national samples, showed acceptable fit, *χ*^*2*^ (1169.56) = 227, *CFI* = .91, *TLI* = .90, *RMSEA* = .05. This suggests that, at least at a basic level, the items functioned in the same manner across these nine nationalities.

Given these analyses, we found support for Hypotheses 2 and 3. Specifically, we established (a) which national samples best fit the three-factor model, and (b) configural invariance, an essential estimate of measurement equivalence (Vandenberg & Lance 2000), of the three factor model across nine of the nationalities. Table 3 presents the means and standard deviations for each of the three factors, separately for each of the nine nationalities. Given that configural invariance was established across these nine nationalities, we can be more confident that scores on the three competencies can be reasonably compared across nine nationalities.

**Table 3:** **Descriptive statistics for GLTAP – three factor model**

	Instilling trust		Respecting beliefs		Navigating ambiguity		Total score	
	*M*	*SD*	*M*	*SD*	*M*	*SD*	*M*	*SD*
Australia	2.81	0.64	3.54	0.80	2.66	0.63	3.00	0.69
Belgium	2.86	0.45	3.18	0.60	2.85	0.57	2.96	0.54
Canada	2.85	0.64	3.43	0.73	2.69	0.62	2.99	0.66
China	2.48	0.47	2.33	0.70	2.11	0.49	2.31	0.55
France	2.60	0.48	2.76	0.61	2.52	0.64	2.63	0.58
Germany	2.76	0.53	2.99	0.70	2.70	0.60	2.82	0.61
India	2.50	0.48	2.90	0.84	2.32	0.52	2.57	0.61
United Kingdom	2.75	0.65	3.51	0.62	2.84	0.60	3.03	0.62
United States	3.49	0.48	3.71	0.62	3.07	0.59	3.42	0.56
Full Sample	2.79	0.62	3.18	0.81	2.64	0.64	2.87	0.69

The most strongly held competency by all nine nationalities was *Respecting Beliefs (3.18)*, followed by *Instilling Trust (2.79)* and then *Navigating Ambiguity (2.64)*. The four figures that follow show how leaders of each of the nine nationalities compared on the competencies identified as being the strongest and consistent across nationalities.

### Total of aggregated competencies

**Figure 3:**
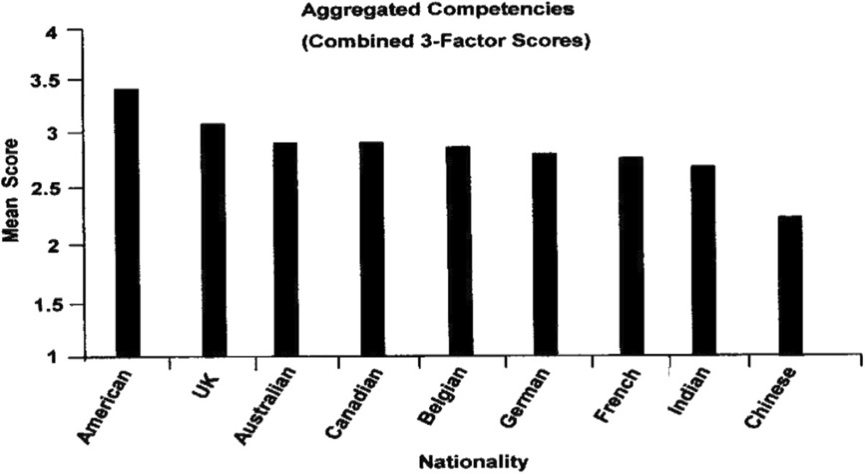
**Aggregated competencies by nationality.**

Overall GLTAP mean scores by nationality are shown in Figure 3. American leaders had the highest mean scores for the aggregated competencies, while the Chinese leaders had the lowest. The other seven national samples had similar mean scores.

### Respecting beliefs

**Figure 4:**
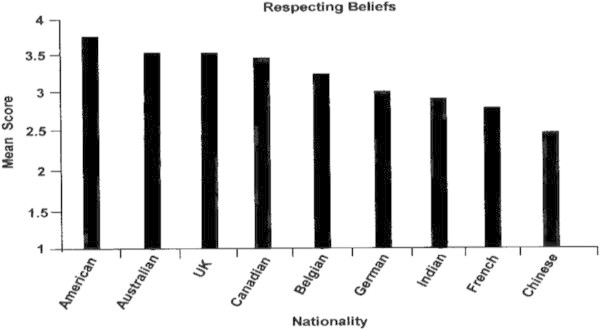
**Respecting beliefs by nationality.**

Again, the American leaders were the strongest in this competency (Figure 4), followed closely by Australian, British and Canadian leaders. The Chinese, French and Indian leaders were the least proficient in this competency.

### Navigating ambiguity

**Figure 5:**
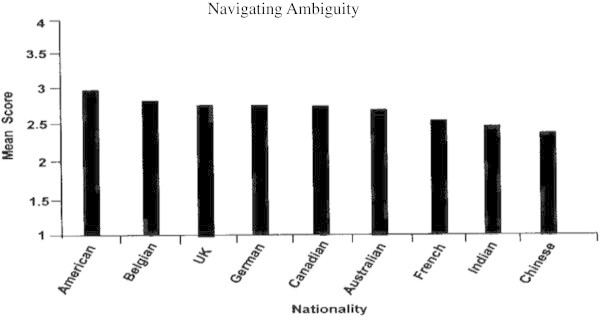
**Navigating ambiguity by nationality.**

The mean scores for this competency were not as differentiated by nationality (Figure 5). However, the Americans again ranked strongest, followed by the Belgians, British and Germans.

#### Instilling trust

**Figure 6:**
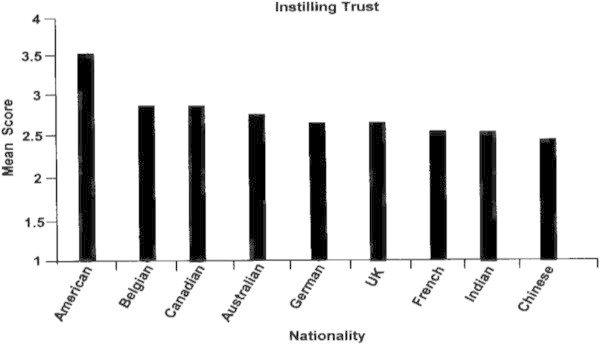
**Instilling trust by nationality.**

The American leaders were far stronger in this competency than any other nationality (Figure 6). Again, the Chinese, Indians and French trailed on the lower end of the mean scores for proficiency.

### Analyses of GBE responses

The Global Business Experience (GBE) survey measures respondents’ self-reported experiences and behaviors concerning global leadership, and was used as the predictive criterion in this study. The GBE was designed to measure three factors: *Global Networking* (α = .78), *Driving Performance* (α = .91), and *Building Team Effectiveness* (α = .97). Six hundred and eighty-nine of the 1469 leaders from the nine nationalities with GLTAP data also completed the GBE, three to six months after completing the GLTAP. A CFA was conducted on these leaders’ data to assess the hypothesized three-factor structure, *χ*^*2*^ (51) = 560.07, *CFI* = .92, *TLI* = .90, *RMSEA* = .12. Although the RMSEA value associated with this analysis indicates less than acceptable fit, both the CFI and TLI produced acceptable fit standards (MacDonald & Ho, 2002), providing support for Hypothesis 4. Descriptive statistics for overall GBE scores (Success Index) and for each of the three GBE factors are presented separately for each nationality and the full sample in Table 4 (Confirmation for hypothesis 5).

**Table 4:** **Descriptive statistics for the GBE across nine nationalities for GBE**

	Global networking		Driving performance		Building team effectiveness		Success index	
Nationality	M	SD	M	SD	M	SD	M	SD
Australia	10.00	6.44	10.02	6.66	10.34	7.05	10.12	6.72
Belgium	11.76	5.11	12.72	4.21	14.11	3.64	12.86	4.32
Canada	11.83	4.6	12.38	4.42	13.08	4.48	12.43	4.50
China	14.39	3.48	14.55	3.08	14.52	3.27	14.49	3.28
France	12.21	4.82	12.74	4.18	13.57	3.93	12.84	4.31
Germany	10.59	5.55	11.42	5.09	12.32	4.93	11.44	5.19
India	12.81	5.24	13.53	4.62	13.77	4.53	13.37	4.80
UK	12.56	5.54	12.23	5.22	13.06	5.43	12.62	5.40
US	13.09	5.8	12.66	5.82	13.8	5.73	13.18	5.78
Total Sample	11.98	5.21	12.35	4.86	13.04	4.84	12.46	4.97

### Measurement equivalence analysis for the GBE

As with the GLTAP data, separate CFAs were conducted for each nationality on the three factor GBE model. The fit statistics for each nationality are in Table 5. Following the CFAs conducted separately for each national sample, a multiple groups CFA was conducted to test for configural invariance across all nine nationalities, *χ*^*2*^ (459) = 1317.86, *CFI* = .88, *TLI* = .85, *RMSEA* = .16. This shows CFI and TLI indices approach acceptable fit standards, while the RMSEA value associated with this model falls well outside of acceptable standards. Taken together, there appears to be configural noninvariance between nationalities (Hypothesis 6).

**Table 5:** **GBE CFAs by nationality**

Nationality	CFI	TLI	RMSEA
Australian	0.90	0.87	0.17
Belgian	0.92	0.90	0.12
Canadian	0.92	0.89	0.12
Chinese	0.86	0.81	0.16
French	0.94	0.92	0.11
German	0.85	0.81	0.17
Indian	0.88	0.85	0.18
UK	0.83	0.78	0.19
American	0.84	0.79	0.15

#### Success factors compared across nationalities

**Figure 7:**
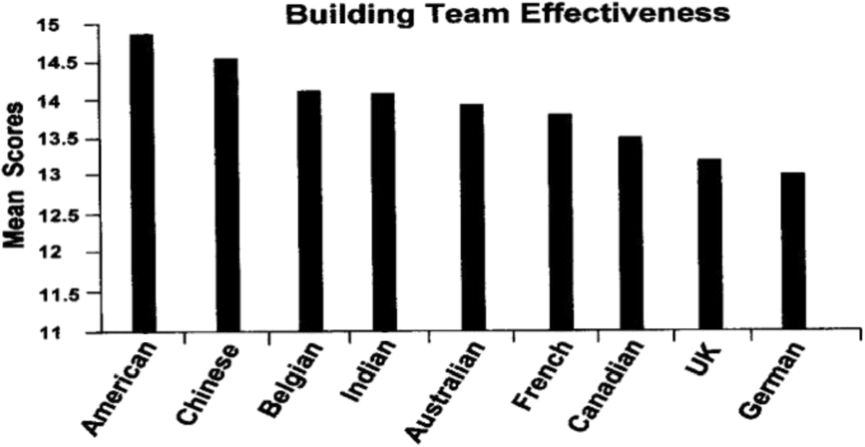
**Building team effectiveness by nationality.**

**Figure 8:**
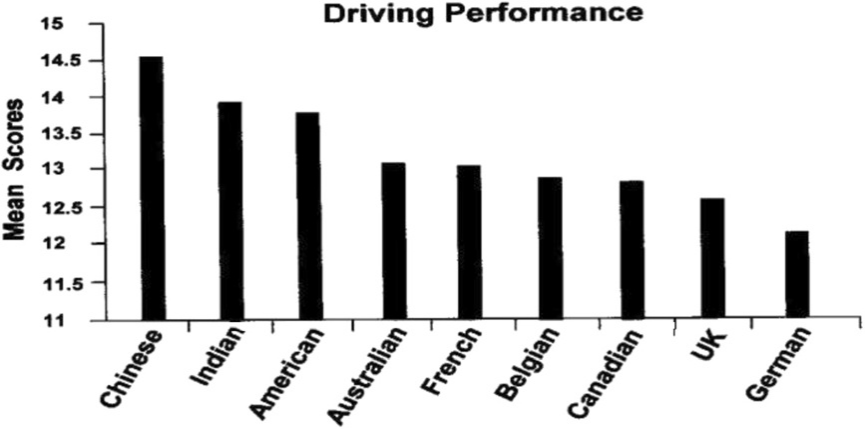
**Driving performance by nationality.**

**Figure 9:**
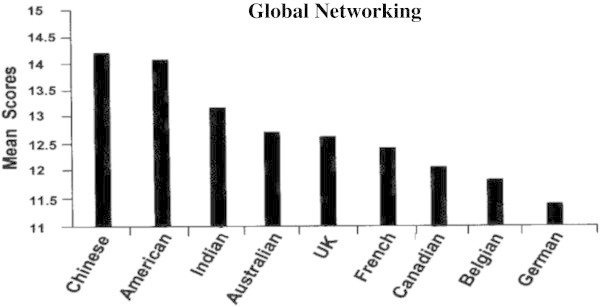
**Global networking by nationality.**

**Figure 10:**
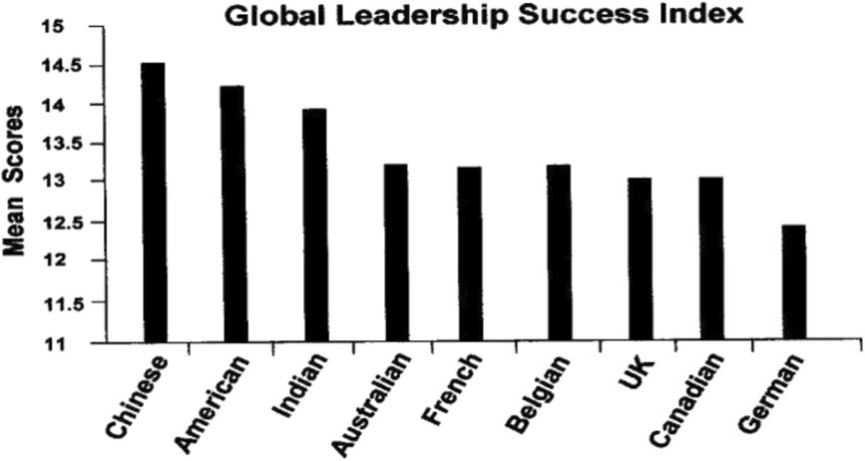
**Global leader success index by nationality.**

Figures 7, 8, 9, 10 show how leaders of each of the nine nationalities compared on the success factors. Note that GBE responses were made using a 0 to 20 scale, but that these figures reflect a smaller portion of the scale in order to illustrate differences.

### Global networking

Interestingly, the rank order of GBE scores by nationality is quite different than those for the GLTAP intercultural competencies. For example, Chinese respondents ranked lowest on each of the GLTAP competencies, and highest on each GBE success factor. Indian and French respondents also ranked higher on GBE success factors than on GLTAP factors. We discuss possible explanations of these differences in rank order in the Discussion section.

### GLTAP intercultural competencies predicting GBE success criteria

#### Multilevel modeling

The initial approach to determine how well the GLTAP competency factors predict the GBE success criteria was to use multilevel modeling (MLM), wherein individual respondents are be nested within nationality. This approach allows both the variance due to individual differences (i.e., within-nationality) and group-based differences (i.e., between-nationality) in the data to be modeled. However, we found that over 96% of the variance in the data was due to within-nationality differences, meaning that only less than 4% of variance in the data was due to differences between nationalities. Because the amount of variance associated with between-nationality differences was minimal, there was little value in the use of MLM over the more widely used and less statistically complex multiple linear regression (MLR) approach. Thus, we proceeded using MLR.

#### Six factor GLTAP

We were unable to support the construct validity of the six-factor GLTAP through CFA. However, the primary use of these six factors is for predicting relevant criteria. Thus, we determined it important to assess the criterion-related validity of all six factors. First, we examined overall GLTAP scores predicting overall GBE scores. R = .22 *R*^2^ = 4.80%, *F*1 = 34.94, *p* < .001, *β* = 24.23, *p* < .001. Next, we included each of the six factor scores as separate predictors, in order to assess the criterion-related validity of separate factor scores when included in a single model. The results of the overall model are as follows: R = .29 *R*^2^ = 8.30%, *F*6 = 10.26, *p* < .001. These results show that the six factor GLTAP model significantly predicted GBE success scores.

#### Extreme groups analysis

Next we assessed the validity of the GLTAP competency factors in predicting the GBE criteria using an extreme groups analysis approach. Twenty percent of those who scored highest (N=141) and lowest on the GBE (N=143) were identified for comparison. Using hierarchical logistic regression, we predicted group membership (high GBE scores/low GBE scores) with respondents’ scores on the three GLTAP factors established through CFA: *Respecting Beliefs, Navigating Ambiguity, and Instilling Trust*. Based on GLTAP factor scores, 55% of those with the highest GBE scores and 54% of those with the lowest GBE scores were correctly identified in the model. We then added the three additional factors from the initially proposed six-factor model: *Adapting Socially, Even Disposition, and Demonstrating Creativity*. The six-factor model correctly classified 67% of those with the top 20% of GBE scores and 73% of those with the lowest 20% GBE scores, a substantial improvement on the three-factor model, *χ*^2^ (6, N = 284) = 49.29, p < .001. These results demonstrate that scores on the six GLTAP factors can be fairly effective at differentiating those with the highest and lowest GBE success scores, and the use of scores on all six proposed factors is more effective at correctly predicting group membership than scores on the three factors established based on CFA. Taken together, findings from predictive analyses provide support for Hypothesis 7.

## Discussion

In the present study we sought to assess the validity of the GLTAP as an measure of global leadership competencies. We failed to find support for the initially proposed model, yet we identified a more parsimonious model demonstrating improved factorial validity. We then identified a subset of nationalities across which evidence indicates the equivalent functioning of the GLTAP factor structure. Next, we validated the factor structure of the GBE across the sample, but failed to demonstrate the equivalent functioning of the criterion measure across nationalities. Nonetheless, we found overall GLTAP and factor scores accounted for a modest amount of variance in GBE success scores, and six GLTAP factor scores showed to be effective in identifying respondents whose GBE success scores were among those in the top and bottom 20% of distribution in our sample. We describe each finding in greater depth below.

This study set out to provide answers to six questions regarding intercultural competencies of global leaders. Discussion of the study is organized here according to these questions.

*What are the intercultural competencies among global leaders?* A set of six competencies emerged: Respecting Beliefs; Navigating Ambiguity; Instilling Trust; Adapting Socially; Even Disposition and Demonstrating Creativity. The alpha reliabilities for these factors indicate that these leaders were grouping sets of items in the GLTAP in somewhat different ways than was hypothesized. Five of the competencies were similar to those constructs that were hypothesized, but the Creativity dimension was a new discovery. Creativity was perceived by these leaders as a combination of Flexibility, Adapting Socially, Lifetime Learning and Navigating Ambiguity. This competency could therefore be viewed as “Social and Situational Creativity”.

The psychometric strengths of these six competencies, and the large sample size comprised exclusively of global leaders, provides confidence that these can be used for global leadership assessment and development, and for further research.

### These factors are described below in terms of leadership competencies

#### Respecting beliefs (RB – 7 items)

This competency represents a leader’s ability to demonstrate respect for the political and spiritual beliefs of people in other cultures. It also includes a good sense of humor, which is an often mentioned but underappreciated, aspect of global leadership. Leaders who can use appropriate humor in tense situations involving political or spiritual differences can diffuse tensions and *loosen things up* for more successful problem solving. This competency focuses on respecting beliefs, which can be very sensitive across cultures, particularly when it comes to politics and spiritual beliefs. Those in global leadership roles must be careful in both verbal and non-verbal messages to not only avoid disrespectful comments, but to learn enough about the beliefs of their people to show respect (such as acknowledging important dates and ceremonies).

While it is essential that global business leaders understand and remain abreast of the political environments in the countries where they operate, they must be sensitive to deeply held political beliefs. The wide gulf in the current American political scene, and the difficulties faced by members of the European Union, present subjects that are rife for passionate debate but would be wise to be avoided by global business leaders.

With respect to spiritual beliefs for example, in Africa a leader must recognize the importance of extended family death rituals and accommodate employee leave times for funerals.

Another example is, in Muslim societies, a leader must adjust organizational life to the five pillars of Islam, including the five daily prayers.

#### Navigating ambiguity (NA 9 items)

This competency represents a leader’s ability to see through vagueness and uncertainty, not become frustrated, and figure out how things are done in other cultures. Ambiguous situations are the norm in leading across cultures, so that the ability to work successfully in these environments is truly an advantage.

One way that leaders express this competency is by avoiding the concept of “Misattribution of Motives and Behavior”. When confronted with foreign ways, people naturally tend to attribute what is seen and heard based one’s own cultural background. Leaders from cultures with a low-context or direct style of communication, for instance, may find the long and circular process of decision-making characteristic of more indirect, high context cultures frustrating and ambiguous. These leaders (mainly from Western cultures) may attribute this to disagreement with their own plan or proposal among their (Asian) counterparts, or to poor decision-making capability. The correct attribution is that longer decision-making for their counterparts is culturally natural for them and involves more stakeholders and leads to quicker implementation.

#### Instilling trust (IT 8 items)

This competency represents a leader’s ability to build and maintain trusting relationships. Extensive research and practice among global teams concludes that trust is the one glue that holds these diverse teams together. Building and maintaining trust across cultures is a complicated process, because trust does not mean the same thing to members of different cultures. Successful global leaders take the time to understand these cultural differences among their people and to build and maintain trust in appropriate ways.

According to Covey and Merrill (2006),

Low levels of trust typically slow down everything—every decision, every communication, and every relationship. On the other hand, high trust produces speed. Leaders who bring high trust to multicultural organizations get superior results by clarifying expectations, listening first, creating transparency and practicing accountability.

#### Adapting socially (AS 12 items)

This competency represents a leader’s ability to socialize comfortably with new people in unfamiliar social situations and to demonstrate genuine interest in other people. Many studies have shown that Adapting Socially is a powerful predictor of intercultural adjustment. Much of global business takes place in social situations, over food and drink, and leaders who can recognize and engage appropriately in these situations are more successful than those who don’t. An important aspect of this competency is showing interest in other people. Remembering and correctly pronouncing names, as well as remembering and repeating things learned about others are ways to do this.

A critical lesson that global businesses have learned in order to succeed in Asia is that networking and relationship building is the essence of Asian business cultures. Leaders who have a high Adapting Socially competence recognize this and are able to do this. This approach is quite different, for example, for Western retailers who want to succeed with sourcing in Asia. They are not used to building relationships with their domestic suppliers, relying instead on requiring the best products at the lowest price. They can do this as well in Asia, but with much more long-term success and loyalty through networking and relationship building with their Asian suppliers.

#### Even disposition (ED 7 items)

This competency represents a leaders ability to remain calm, not being critical of oneself and learning from mistakes. In good times and especially in bad, people in an organization look to their leaders for guidance. Those leaders who take things in stride and maintain an even disposition set a tone for the organization culture that is resilient.

#### Demonstrating creativity (DC 8 items)

This competency represents a leaders ability to enjoy new challenges, strive for innovative solutions to social and situational issues and to learn from a variety of sources. This quality includes the ability to see around corners, predict outcomes and act despite uncertainty. This dimension of creativity is therefore related to the Navigating Ambiguity dimension discussed above. Creative approaches are more difficult, but more successful, in ambiguous situations.

Creative global leaders practice and encourage experimentation and innovation throughout their organizations. They expect to make deeper business model changes to realize their strategies, take more calculated risks, find and support new ideas, and keep innovating in how they lead and communicate. Successful global leadership is all about leading through others, finding creative ways to select, retain and motivate diverse talent. It is also about maintaining a competitive, creative edge through lifetime learning—making a habit of learning from a variety of sources.

Among these factors, three did not function as predicted, and we failed to find support for the six-factor model described above. However, we did find some support for the factorial validity of a model that included a subset of these factors. Nonetheless, each of these factors was further examined through criterion-related analyses to assess their practical utility.

##### What are the strongest of these competencies and those in need of greatest development?

Equivalence analyses showed that three of the competencies could be compared across cultures. Of these, Respecting Beliefs was the strongest. This is encouraging, given the volatile situation involving spiritual and political differences in the world today. Leaders who can maintain a focus on organizational issues, while demonstrating an understanding and respect for the diversity of beliefs in his or her global organization, are certainly in great need. An interesting aspect of this competency was a value on the use of humor to diffuse tense or stressful situations. This is an often overlooked aspect of successful leadership, but one that is quickly recognized and appreciated in those leaders who use humor appropriately.

Instilling Trust and Navigating Ambiguity were rated lowest of the three competencies. This is not surprising, given the complexity of trust and its meaning across cultures, and the fact that global business operates in much more of a grey area than black-and white.

A total score of all three competencies had a mean of only 2.86 on a five-point scale, indicating that these leaders have some way to go for full development of these competencies. These results support the surveys and studies cited in the Introduction to this article, that global organizations do not have the leaders needed to keep up with the speed of business, are not satisfied with the quality of their leaders, and do not have the bench strength to meet future business needs. This study points to specific areas to meet these needs.

##### How do these competencies differ across nationalities?

The American leaders had the highest mean scores for the aggregated list of competencies, followed by the British and Australian leaders, while the Chinese leaders had the lowest with the French and Indian leaders trailing close behind.

Explanations for these differences can only come from an in-depth look at each nationality, its business culture, and how these differ from one another. One is issue may be that Chinese, French and Indian leaders have different understandings of the meaning associated with these competency factors than leaders of other nationalities. However, several useful and logical explanations follow. It may be that the American, British and Australian (the three nationalities with the highest GLTAP overall mean scores) business cultures have been greatly affected in recent years by initiatives in the areas of inclusion and cultural awareness. Canadians (fourth highest GLTAP overall mean score) celebrate multiculturalism and it is a source of national pride. Also, leaders in these four cultures have a fairly long history of working in multinational businesses, while those in China and India are quite new to this. Chinese leaders are more experienced with leading Chinese state-owned enterprises than they are with multinational companies. Indian leaders are more experienced with leading large family-owned businesses and Indian state-owned businesses than they are with multinational companies.

The Chinese, Indians and French trailed on the lower end of the mean scores for the Navigating Ambiguity proficiency. The Chinese culture is high on “uncertainty avoidance”, (Hofstede, 1997) which is not surprising given the country’s history of authoritarian rule. Therefore, any areas of ambiguity need to be clearly laid out for them with specific steps and actions in how to get through it. This may partially explain why the Chinese scored the lowest on the Navigating Ambiguity competency.

Differences in the competency of Instilling Trust by nationality may be partially explained by the concept of “tight” and “loose” cultures. Gunia, Brett, Nandkeolyar & Kamdar (2011); Yamagishi and colleagues (Takahashi et al. 2008;Yamagishi et al. 1998;Yamagishi & Yamagishi 1994). Tight cultures are those in which social norms are clearly defined and reliably imposed, leaving little room for improvisation or interpretation. Loose cultures are those in which social norms are flexible and informal. They propose expectations but permit individuals to define the range of tolerable behavior within which they may exercise their own preferences. Thus, enforcement in loose cultures is left to interpersonal mechanisms. According to Gunia, et al. (2011) this concept may be applied to trust as follows:

“Because institutional mechanisms govern behavior in tight cultures, individuals from these cultures tend to rely on institutional trust more than interpersonal trust to control behavior and sanction deviance. Because interpersonal mechanisms govern behavior in loose cultures, the exact opposite is true.” Yamagishi (2009) asserted that people in cultures with strong social norms “do not need social intelligence to find out who is trustworthy—trust is not needed”.

Applying this concept to the differences among national leaders with respect to the Instilling Trust competency, it is expected that the Chinese and Indians, and to some extent, the French, who all scored low on Instilling Trust, would represent tight cultures, while the Americans, Belgians, Canadians and Australians, who scored high on Instilling Trust, would represent loose cultures. Gelfand MJ, Raver J, Nishii L, Leslie K, Lun J, Lim BC, Yan X. The difference between “tight” and “Loose” societies revisited: Ecological, social-political, and societal correlates of tightness-looseness in modern nations. Manuscript submitted for publication, presented tightness scores for thirty-three nationalities, including the following (the higher the score, the more “tight” the culture):

- Indian = 11
- Chinese = 7.9
- French = 6.3
- American = 5.1
- Belgian = 5.6
- Australian = 4.4

These tightness/looseness scores may therefore mean that the Indian leaders especially, and also the Chinese and French, have lower competencies on Instilling Trust because they represent tight cultures as compared with the loose cultures of the Americans, Belgians and Australians. The Indian, Chinese and French leaders rely more on assumed norms regarding trust, while the American, Belgian and Australian leaders take individual action, responsibility and accountability to instill trust among their people. These findings have an important message for the Indian, Chinese and French leaders who are leading multicultural organizations. They need to learn what trust means among the cultures of their people, and spend time and energy to develop trust as a central, expressed value in their organizations.

##### What are the areas of greatest leadership success?

Factor structure supported a three factor model (Hypotheses 3). However, we failed to establish configural invariance (Hypothesis 4), suggesting that these factors may reflect different constructs across nationalities. This evidence impairs the factorial validity of the GBE, yet there are multiple types of validity (see Messick 1989), and in the case of the GLTAP and GBE it could argued that whether they work (criterion-related validity) may be more important than how they work (factorial validity). Thus, we subsequently used the GBE to test the criterion-related validity of GLTAP factor scores.

The highest rated success score was for Building Team Effectiveness. One thing that is very clear about the global business environment is that everyone works in one or more teams, either on a virtual basis or face-to-face. A large body of work has emerged to support this teamwork (Larson & LaFasto 1989;LaFasto & Larson 2001;Ray & Bronstein 1995;Parker 1994). Apparently, the leaders in this study have benefitted from this attention to team effectiveness and rate themselves relatively high on their ability to lead global teams.

Similar to the finding for intercultural competencies, a total score on all three factors of leadership success had a mean of only 13.33 on a 20-point scale, indicating that these leaders see significant room for improvement. Also, the psychometric strengths of these three factors, and the large sample size comprised exclusively of global leaders, provides confidence that these can be used as criterion measures for global leadership assessment and development and for further research.

##### How do these success factors differ across nationalities?

Equivalence analyses showed some evidence that this three-factor model could be compared across nationalities. Comparisons should be considered as general relative rankings, therefore, before we cannot be sure that leaders from these countries interpreted GBE items in the same manner as respondents from other countries. Overall, the Chinese had the highest mean scores, followed by the Americans, Indians, Australians and French. The rank order pattern of these success factors is quite different from that for the intercultural competencies. The Chinese had ranked lowest of the nine nationalities on the competencies, and highest on the success factors. The Indians and French also ranked higher on these success factors. One reason behind this might be that those nationalities that ranked lower on the global competencies and higher on the self-rated success factors may not have a realistic handle on the outside world’s perception of them and the reality of their own performance. Also, the German leaders may have scored lowest on these success factors because of their tendency not to overstate their accomplishments, and to focus very specifically on the metrics of the GBE instrument.

A possible interpretation of this phenomenon might be found in Hofstede’s Power Distance concept (Hofstede 1997). Power Distance is defined as:

*The degree to which inequality or distance between those in charge and the less powerful (subordinates) is accepted in a culture.*

A high Power Distance culture favors a leadership style that is hierarchical, while a low Power Distance culture favors a participative style. Table 6 compares the success rankings with the relative rankings of these nine nationalities on Power Distance. (These are not Hofstede’s original rankings. They are the relative rankings of these nine nationalities, based on the Hofstede data).

**Table 6:** **Power distance and leadership success**

Power distance rankings	Leadership success rankings
(Large to Small)	(High to Low)
1. China	1. Chinese
2. India	2. American
3. France	3. Indians
4. Belgium	4. Australian
5. USA	5. French
6. Australia	6. Belgian
7. Canada	7. British
8.5. Great Britain	8. Canadian
8.5. Germany	9. German

A highly significant rank order correlation was found between these two sets of rankings (r = .804), indicating that the larger Power Distance nationalities were also ranking higher on leadership success factors, and vise-versa (with the notable exception of the Americans). It may be that a characteristic of large Power Distance (hierarchical) leaders is to consider themselves and their organizations as more successful than small Power Distance (participative) leaders. They have achieved the highest levels of their organizations and may be less aware of their subordinates and others’ view of their leadership success.

A final comment here is that our two instruments were completed in English and the items were not subjected to cross-language/culture adjustments for construct, method and item bias. However, we think that the clear differences on the competency and success factors were probably due to Power Distance and tight/loose cultures and not that some nationalities are less familiar with psychometric instruments. One would conclude that if they are less familiar with psychometric instruments, their results would be random, and not display the statistical differences we found.

##### Which competencies are most strongly predictive of leadership success?

**Figure 11:**
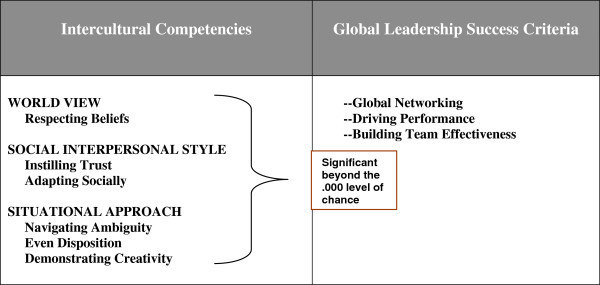
**Modified transcultural leadership model.**

The six factor GLTAP model significantly predicted GBE success scores. Further, when those in the top and bottom 20% of the distribution of GBE scores were selected for extreme group analyses, both the three-factor and six-factor GLTAP models were significant predictors of group membership (top 20%/bottom 20% of GBE scores), and despite our failure to find evidence supporting the factorial validity for the six-factor model, it demonstrated a substantial improvement over the three-factor model in terms of predicting group membership. Thus, the six-factor model may have greater practical utility than three-factor model. The predictive model is shown in Figure 11, which is also our modified Transcultural Leadership Model.

### Contribution to leadership theory

**Figure 12:**
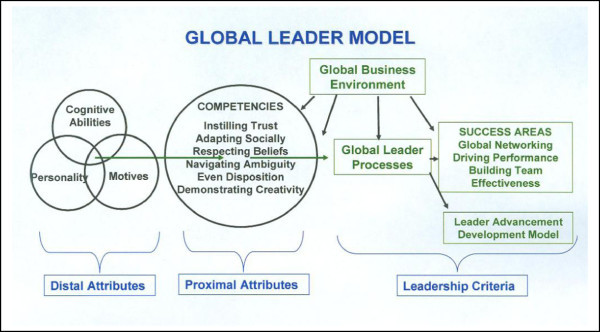
**Global leader model.** Adapted from: Zaccaro 2007.

As described earlier, a goal of this study was to apply Zaccaro’s (2007) trait-based model of leader effectiveness to the context of global leadership and specify a set of global leadership-relevant proximal attributes. This study identified six potential intercultural competencies as proximal attributes of effective global leaders. These intercultural competencies are influenced by leader traits, and both leader traits and competencies are theorized to affect leader processes. In addition, the global business environment within which the leader is operating is believed to directly influence intercultural competencies and leaders’ processes, as well as the competency-process and process-criteria relationships (see Figure 12).

We are therefore stating the following theoretical proposition regarding successful global leadership:

- *Distal Attributes:* successful global leaders most certainly have a high level of cognitive abilities; perhaps some personality traits such as extroversion, curiosity and relationship management; and a set of motives and values that drive a personal interest in other cultures.
- *Proximal Attributes:* we have identified six measurable intercultural competencies which interact with distal attributes in important ways that lead to successful global leader performance. For example, a leader with a high level of cognitive ability without the competency of dealing with intercultural ambiguity would not be very successful in the global business environment.
- *Leader Effectiveness:* we have identified three measurable global leader success factors that comprise leader effectiveness in the global business environment.

### Limitations, further research, and practical implications

As with all research, there are a number of limitations associated with this study. First, both the GLTAP and the GBE were completed in English. Although all respondents were senior leaders in positions requiring interaction with foreign cultures and all leaders indicated English as either a primary or secondary language, leaders’ proficiency with English was unknown. This may have led to the misinterpretation of GLTAP and/or GBE items, or the inability to interpret items at all. Consequently, this may have led to increased measurement error within the data and negatively affected our attempts to establish the factorial validity of the GLTAP and GBE. For example, our failure to establish configural invariance in the GBE across nationalities may have resulted from measurement error due to a English language difficulties among some participants. Further attempts to validate the GLTAP and GBE may find it useful to: assess respondents’ English proficiency with a single question (e.g., “Please rate your English using the following scale”), ensure respondents used in future analyses possess adequate proficiency with the English language, or provide versions of the measure in respondents native language.

Second, this study lacked the statistical power necessary to fully assess the factorial validity of the GLTAP. With regard to the GLTAP, larger samples will be necessary in order to conduct meaningful measurement invariance analyses in the future, and as the use of the GLTAP becomes more widespread sufficient samples to conduct these and other validity analyses will become possible.

These findings provide important practical implications. This study has resulted in a set of intercultural competencies that may be used for global talent assessment and development. Guidelines for applying these results are described here in the following applications: assessment, feedback and development.

### Intercultural competency assessment

The GLTAP can be accessed and completed on-line. It is scored and interpreted in comparison to the global leader sample as well as the nationality of the respondent. All six competencies measured by the GLTAP can be used and interpreted as initial indications of relative position on these competencies. They should be further investigated and verified by means of the Behavioral Interview technique. The assessment process is continued with Certified Assessors conducting a Behavioral Interview, based on the GLTAP Profile. The basic assumption underlying this type of interview is that *the best predictor of future behavior is past behavior*. Therefore, the Assessor elicits specific examples to verify the TAP results. This lead-in question could be asked for Navigating Ambiguity: *Some people seem to enjoy and do well in situations that are unstructured or unclear—would this characterize you?* A follow-up behavioral question might be: *Give me a specific instance in which you found yourself in this kind of situation---unclear rules or guidelines, not all sure what should be done. Describe the situation, what you did and how it worked out.* Depending on the quality of the responses to these behavioral questions, the Assessor makes a final qualitative judgment of competency strengths and development areas.

### Coaching for intercultural competency feedback and development

Following behavioral verification, the feedback and development process begins. A Feedback Guide is presented and the GLTAP Profile is explained. Examples given during the interview are used to better explain strengths and areas for development. A goal here is for the respondent to take ownership for his or her Profile and to understand its value in becoming a better global leader. A Development Plan is then presented and specific actions are agreed upon. Following this initial feedback and development phase, on-going coaching is planned and the process is integrated into the respondent organization’s overall talent development program.

## Conclusions

The present study provides an initial attempt to validate the GLTAP and GBE. Sufficient factor structures were established for both measures. GLTAP factor scores accounted for a modest amount of variance in GBE scores across the entire sample, and the six factor scores of the GLTAP, together, appear to be effective at differentiating those who self-reported being the most successful global leaders from those who reported the lowest global leadership success scores. This paper also describes the role of the GLTAP and GBE within a broader global leadership development program.

Successful global leaders therefore emerge from this research as those who:

- Enjoy new challenges, strive for innovative solutions to social and situational issues and learn from a variety of sources;
- Build and maintain trusting relationships;
- Socialize comfortably with new people in unfamiliar social situations, demonstrate genuine interest in other people; and exhibit a good sense of humor
- See through vagueness and uncertainty, do not become frustrated, and figure out how things are done in other cultures;
- Remain calm, without being critical of oneself
- Demonstrate respect for the political and spiritual beliefs of people of other cultures.
